# Transcutaneous vagus nerve stimulation: a new strategy for Alzheimer’s disease intervention through the brain-gut-microbiota axis?

**DOI:** 10.3389/fnagi.2024.1334887

**Published:** 2024-02-27

**Authors:** Long Yan, Hong Li, Yulin Qian, Junfeng Zhang, Shan Cong, Xuemin Zhang, Linna Wu, Yu Wang, Meng Wang, Tao Yu

**Affiliations:** ^1^The First Teaching Hospital of Tianjin University of Traditional Chinese Medicine, Tianjin, China; ^2^National Clinical Research Center for Acupuncture and Moxibustion, Tianjin, China; ^3^Graduate Department, Tianjin University of Traditional Chinese Medicine, Tianjin, China

**Keywords:** transcutaneous vagus nerve stimulation, Alzheimer’s disease, brain-gut-microbiota axis, HPA axis, microbiota

## Abstract

Transcutaneous vagus nerve stimulation (tVNS) is an emerging non-invasive technique designed to stimulate branches of the vagus nerve distributed over the body surface. Studies suggest a correlation between the brain-gut-microbiota (BGM) axis and the pathogenesis of Alzheimer’s disease (AD). The BGM axis represents a complex bidirectional communication system, with the vagus nerve being a crucial component. Therefore, non-invasive electrical stimulation of the vagus nerve might have the potential to modify—most of the time probably in a non-physiological way—the signal transmission within the BGM axis, potentially influencing the progression or symptoms of AD. This review explores the interaction between percutaneous vagus nerve stimulation and the BGM axis, emphasizing its potential effects on AD. It examines various aspects, such as specific brain regions, gut microbiota composition, maintenance of intestinal environmental homeostasis, inflammatory responses, brain plasticity, and hypothalamic–pituitary–adrenal (HPA) axis regulation. The review suggests that tVNS could serve as an effective strategy to modulate the BGM axis and potentially intervene in the progression or treatment of Alzheimer’s disease in the future.

## 1 Introduction

Alzheimer’s disease (AD), a leading cause of dementia, accounts for 60–70% of all dementia cases. It is projected to affect 150 million people globally by 2050 (Alzheimer's Association, 2023), positioning it as a major public health challenge (Alzheimer's Association, 2022). AD imposes a heavy burden on families and society. Characterized by cognitive dysfunction, impaired learning abilities, reduced executive function, and memory decay (Zvěřová, 2019; Knopman et al., 2021), its pathology involves neurofibrillary tangles due to abnormal accumulations of Aβ amyloid and hyperphosphorylated tau proteins, leading to associated neurological loss and neurodegeneration (Chen and Mobley, 2019).

Risk factors influencing AD development include age, genetics, psychiatric conditions, primary brain lesions, systemic comorbidities, and social factors (Devi, 2023). The primary therapeutic approach to AD has been pharmacological, with only 10 FDA-approved treatments to date, including memantine, which is the newest treatment option despite having been available for a decade (Cummings et al., 2014). In the European Union, four treatments are approved for AD: three cholinesterase inhibitors (donepezil, galantamine, and carboplatin) and the N-methyl-D-aspartate receptor antagonist, memantine (Cummings et al., 2019). Despite their widespread clinical use, the efficacy of these drugs is questionable, and they are associated with various side effects (Birks and Harvey, 2018; Folch et al., 2018; Teng et al., 2022). Therefore, exploring new and effective interventions for AD treatment is imperative.

The brain-gut-microbiome (BGM) axis has emerged as a significant area of research in AD treatment and is seen as a potential therapeutic target (Kim et al., 2020). The BGM axis is a bidirectional communication system between the brain and gut (O'Mahony et al., 2011), primarily involved in maintaining dynamic equilibrium in the gastrointestinal tract. It regulates processes related to behavior and brain activity, including higher cognitive functions, motivation, and emotion. This regulation is facilitated through a complex messaging system involving the central nervous system (CNS), autonomic nervous system (ANS), enteric nervous system (ENS), and the hypothalamus-pituitary–adrenal (HPA) axis (Varesi et al., 2022). Recent studies have established a link between BGMA and the pathophysiology of neurodegenerative disorders (Doifode et al., 2021). Despite these advancements, the exact mechanisms by which BGMA influences these disorders remain unclear.

The vagus nerve (VN), an essential component of the parasympathetic nervous system, is the longest in the body, extending from the medulla to the gastrointestinal tract (Dolphin et al., 2022). It is crucial for exchanging information between the brain and various visceral organs. The term ‘vagus’ originates from the Latin ‘vagari’, meaning “to wander” (Butt et al., 2020). The vagus nerve is made up of 20% efferent fibers and 80% afferent fibers. The afferent fibers transmit signals from peripheral organs to the CNS, while efferent fibers transmit signals from the CNS to peripheral organs. These fibers modulate functions in organs such as the heart, lungs, and gastrointestinal tract, regulating anti-inflammatory reflexes, secretion, and peristalsis in the gastrointestinal tract (Bonaz et al., 2018). Anatomical studies have shown that the VN projects centrally to subcortical brain structures including the cerebellum, thalamus, and hippocampus (Yakunina et al., 2018; Trifilio et al., 2023), exerting excitatory effects on these neurons (Rutecki, 1990). The VN transmits signals through four nuclei in the CNS, namely the nucleus tractus solitarius (NTS), the spinal trigeminal nucleus, the solitary nucleus and the dorsomedial nucleus (DMN; Berthoud and Neuhuber, 2000). Notably, the NTS is closely associated with brain regions associated with neurodegenerative diseases, such as the blue patch and the hippocampus (Olsen et al., 2023) and is believed to influence cognitive functions (Humphrey et al., 2023). Therefore, the central connectivity of the VN with brain regions such as the hippocampus provides a neuronal basis for pathophysiological processes linking gut and brain as caused by AD.

Vagus nerve stimulation (VNS), initially used as a treatment by James Leonard Corning (1855–1923) in 1871, was abandoned in the late 19th century due to its lack of therapeutic efficacy. It resurfaced in the 20th century as a treatment for epilepsy, with human trials for implanted VNS devices beginning in 1988. While VNS showed modest benefits in reducing seizure frequency for intractable partial seizures, it also produced side effects like bradycardia, dizziness, and syncope (Lanska, 2002). To mitigate these side effects, unilateral VN stimulation technology was developed in the early 21st century. This innovation received FDA approval for use as an adjunct therapy in refractory epilepsy and depression (O'Reardon et al., 2006). However, traditional VNS methods can cause adverse effects such as vocal cord damage, arrhythmia, surgical risks, including wound infection and sensory abnormalities (Fahy, 2010), and increased costs related to surgery and equipment (Baig et al., 2022). In response, non-invasive vagus nerve stimulation was proposed at the turn of the century (Ventureyra, 2000). It is based on the principle of stimulating vagus afferent fibers using electrodes placed on the skin where the VN is distributed. The primary stimulation sites are the auricular tegmental area (transcutaneous auricular VNS; taVNS) and the neck (transcutaneous cervical VNS; tcVNS; Baig et al., 2022).

Functional magnetic resonance imaging (fMRI) studies have shown that transcutaneous vagus nerve stimulation (tVNS) activates brain regions similar to those stimulated by VNS, including the trigeminal nucleus, the locus coeruleus (LC), the NTS, the amygdala, and the spinal amygdala (Frangos et al., 2015). taVNS, the most common form of tVNS, has been applied to treat various clinical conditions, including epilepsy, depression, migraine, constipation, insomnia, tinnitus, Parkinson’s disease, cognitive disorders, and stroke (Hilz, 2022; Zhang S. et al., 2022). Systematic reviews have confirmed the efficacy of tVNS in treating epilepsy, depression, and migraine reviews (Wu et al., 2018, 2020; Song et al., 2023; Tan et al., 2023). While tVNS has demonstrated potential in treating tinnitus and aiding stroke rehabilitation, its clinical relevance requires further investigation (Yan et al., 2022; Fernández-Hernando et al., 2023).

Previous research has established a connection between vagal activity and hippocampal tissue health. Decreased vagal activity may negatively affect hippocampal tissue. Additionally, electrical stimulation of the VN can influence hippocampal function at both electrophysiological and epigenetic levels. This influence is mediated internally through septal cholinergic mediation (Broncel et al., 2018), promoting synaptic plasticity and improving cognitive function (Olsen et al., 2022).

In recent years, tVNS has gained attention as a potential intervention for AD. A meta-analysis concluded that taVNS positively impacts cognitive function in healthy subjects, particularly in enhancing executive function (Ridgewell et al., 2021). Furthermore, an observational study by Thakkar et al. (2020) found that taVNS improves adult reading proficiency, significantly increasing letter spelling and decoding skills. These findings suggest broader implications for cognitive tasks and highlight the potential of tVNS as a therapeutic tool for cognitive enhancement and treatment of cognitive impairments. According to the review of Vargas-Caballero et al. (2022), VNS (including tVNS) may enhance neuroplasticity and improve LC function to some extent. Additionally, it may modulate neuronal circuits and neuroinflammation.

A randomized controlled trial (RCT) involving patients with mild cognitive impairment (MCI) demonstrated significant cognitive improvements following taVNS intervention. These improvements were measured using cognitive scales such as the Montreal Cognitive Assessment, Pittsburgh Sleep Index, and Boston Naming Test. Wang et al. (2022) concluded that taVNS might be an effective method for preventing or delaying the onset of AD. Kaczmarczyk et al. (2017) reported that tVNS could induce a transformation in microglia phenotype in an AD mouse model, leading to neuroprotective effects. This finding underscores the potential of tVNS treatment for AD. In a study by Yu et al. (2023), taVNS enhanced spatial memory and learning in APP/PS1 mice and decreased the expression of Aβ-41, IL-1β, and IL-18 in microglia through the P2X7R/NLRP3/Caspase-1 signaling pathway. Additionally, taVNS treatment significantly improved memory performance for new object recognition and memory persistence in mice (Vázquez-Oliver et al., 2020). taVNS has also been shown to alleviate cognitive impairments in postoperative rats with cognitive deficits, potentially due to the stimulation of cholinergic anti-inflammatory pathways in the basal forebrain and hippocampal regions. Zhou et al. (2023) observed significant reductions in apoptotic and necrotic proteins, including cleaved caspase-3 and p-MLKL, in the hippocampus and a decrease in activated microglial cell activity. Neurodestructive microglia were converted into a neuroprotective phenotype, improving central anti-inflammatory effects (Kaczmarczyk et al., 2017). Based on these findings, the present evidence proposes that tVNS, particularly taVNS has considerable potential for ameliorating cognitive impairment; nevertheless, the precise mechanisms necessitate further investigation. The BGM axis—in contrast to mere CNS effects—may be an important system through which VNS can affect neurodegeneration. This review explores the relationship between percutaneous VNS and the BGM axis, focusing on its potential impact on AD. It examines the efficacy of tVNS in treating AD through the regulation of signaling within the BGM system, aiming to establish it as a new therapeutic technique for this condition.

## 2 The relationship between NTS dysfunction and AD

The NTS is a vital brainstem multi-signaling hub, serving as a major integration site for central sensory afferents, and is closely related to various neural and hormonal systems (Canessa et al., 2011). Its significance extends to the neuropathogenesis of AD, where it plays a critical role in facilitating the disease’s cascading responses. This is primarily due to the synergistic effects of neuroinflammation and intermittent hypoxia, which result in neuronal and synaptic dysfunction within the NTS. Such dysfunctions have downstream impacts on pathways associated with the NTS, ultimately contributing to cognitive deficits across the CNS (Daulatzai, 2012).

The volume of the NTS has been observed to decrease with age (Celle et al., 2009). Furthermore, lipopolysaccharides (LPS) derived from the gut stimulate the expression of CNS inflammatory factors. The NTS serves as a cytokine detector within the CNS, and the local synthesis of inflammatory mediators directly influences neuronal functionality in the NTS (Dantzer, 2004). In older individuals, apnea can induce hypoxia, thereby increasing the expression of inflammatory cytokines (such as TNF-α, IL-1β), chemokines (IL-8, MCP-1/CCL2), and adhesion molecules (ICAM-1) in astrocytes and brain endothelium. These inflammatory responses can lead to neuron apoptosis in the NTS, further exacerbating its pathology (Zhang et al., 2010). In the context of AD, VNS emerges as a critical therapeutic intervention. VNS may improve NTS pathology through its anti-inflammatory effects and by directly stimulating the NTS. By mitigating NTS dysfunction, VNS potentially exerts a beneficial influence on the progression and symptoms of AD, providing a novel approach to managing this complex neurodegenerative disorder.

## 3 Relationship between the vagus nerve and the gut

The VN plays an essential role in the bidirectional information exchange within the BGM axis, acting as the primary neural conduit between the ENS and the CNS. It mediates the transmission of signals between the brain and gut microbiota, underscoring the interrelation of these systems (Forsythe et al., 2014). Interestingly, research has shown that the VN is pivotal in how the gut microbiota can influence brain function. A study involving *Lactobacillus* strains demonstrated that these bacteria could affect the mRNA expression of GABA (Aα2) receptors in the prefrontal cortex (PFC), amygdala, and hippocampus. However, this effect was not observed in control mice that had undergone a subdiaphragmatic vagotomy (SDV; Bravo et al., 2011). Another study found that SDV could prevent depressive-like behavior and gut microbiota dysbiosis in mice treated with LPS, further supporting the role of the VN in the BGM axis (Zhang et al., 2020). Preclinical studies have recognized Subdiaphragmatic VN as a crucial pathway for information transmission within the BGM axis. It is a primary channel through which the gut microbiota exerts its influence on the CNS (Wang et al., 2023; Yang et al., 2023). Notably, SDV can effectively disrupt the impact of gut microbes on the CNS and cognitive functions (Wu Y. et al., 2021; Yue et al., 2023). These findings highlight the significant function of VN fibers in facilitating communication between the gut microbiota and the brain (Margolis et al., 2021).

The efferent control of gastrointestinal (GI) motility involves direct synapses formed by the VN with intestinal epithelial cells and enteric glial cells (EGCs) in both the mucosal and myenteric layers (Cryan et al., 2019). Although epithelial cells lack sensory innervation, afferent information flows through EGCs (Bonaz, 2022). EGCs are hypothesized to facilitate gut-brain communication via the VN, playing a significant role in regulating gut permeability (Neunlist et al., 2008). Research by Costantini et al. (2010a) reported that EGCs are vital in controlling intestinal permeability through VNS, mediating information exchange between the CNS and ENS. EGCs can form synapses with the VN in the gut epithelium, thereby facilitating CNS with information on changes in gut microbiota structure and function (Kaelberer et al., 2018). Additionally, EGCs are essential for identifying intestinal microbes and their derivatives via Toll-like receptors, thereby modulating GI motility and secretion through vagal afferent fibers (Abreu et al., 2005; Bonaz et al., 2018).

**Figure 1:**
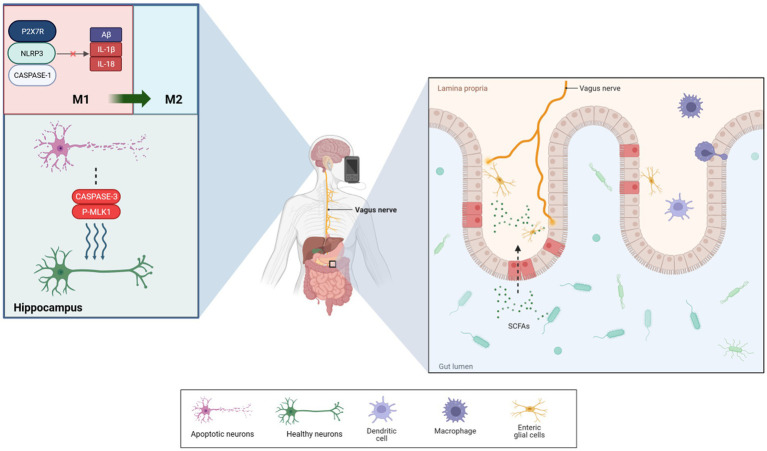
taVNS reduced the levels of Aβ-41, IL-1β, and IL-18 produced by microglia via P2X7R/NLRP3/Caspase-1. It also significantly reduced the levels of apoptotic and necroptotic proteins, which induced microglial phenotypic transformations to protect neurons. Additionally, taVNS improved intestinal permeability, enhancing the diversity of species in the microbiota. Furthermore, enteric glial cells assisted in the exchange of information between the vagus nerve and gut.

To elucidate that neural information does not end at the gut’s wall, information can also be picked up and transmitted from the microorganisms producing chemical signals inside the lumen, it can be found that the vagus nerve can directly detect microbial signals from the gut, such as lactobacilli and short-chain fatty acids, and transmit them to upstream nociceptors (Bravo et al., 2011; Goswami et al., 2018; Wang et al., 2020). Rei et al. (2022) suggested that gut microbiota negatively impacts hippocampus-dependent memory via a vagal-dependent mechanism, potentially contributing to cognitive impairment in dementia. VNS has shown effectiveness in protecting rat intestinal epithelial cell glycoconjugates through cholinergic anti-inflammatory pathways, reducing intestinal barrier permeability by 43% (Wu J. et al., 2021), and modulating norepinephrine concentration levels to influence blood flow in damaged intestines (Yagi et al., 2020). Furthermore, VNS has been observed to improve intestinal barrier integrity, increasing intestinal villus height and expression of tight junction proteins (Costantini et al., 2010b). A study on adolescents with irritable bowel syndrome showed significant improvements in gut microbiota diversity following taVNS (Bora et al., 2023). However, these findings have not been universally replicated. Additionally, VN stimulation has been observed to suppress pro-inflammatory M1 macrophage expression, demonstrating its capacity to modify gut microbiota and intestinal permeability (Yuan and Taché, 2017). As summarized in Figure 1, these mechanisms demonstrate how taVNS can regulate brain and gut functions, suggesting its potential in managing AD. The VN’s role in information transmission between the gut and brain highlights its importance in AD treatment and prevention. Proper stimulation or regulation of the VN function is increasingly advocated for treating and preventing the onset of AD (Décarie-Spain et al., 2024).

## 4 Anti-inflammatory pathway of tVNS

**Figure 2:**
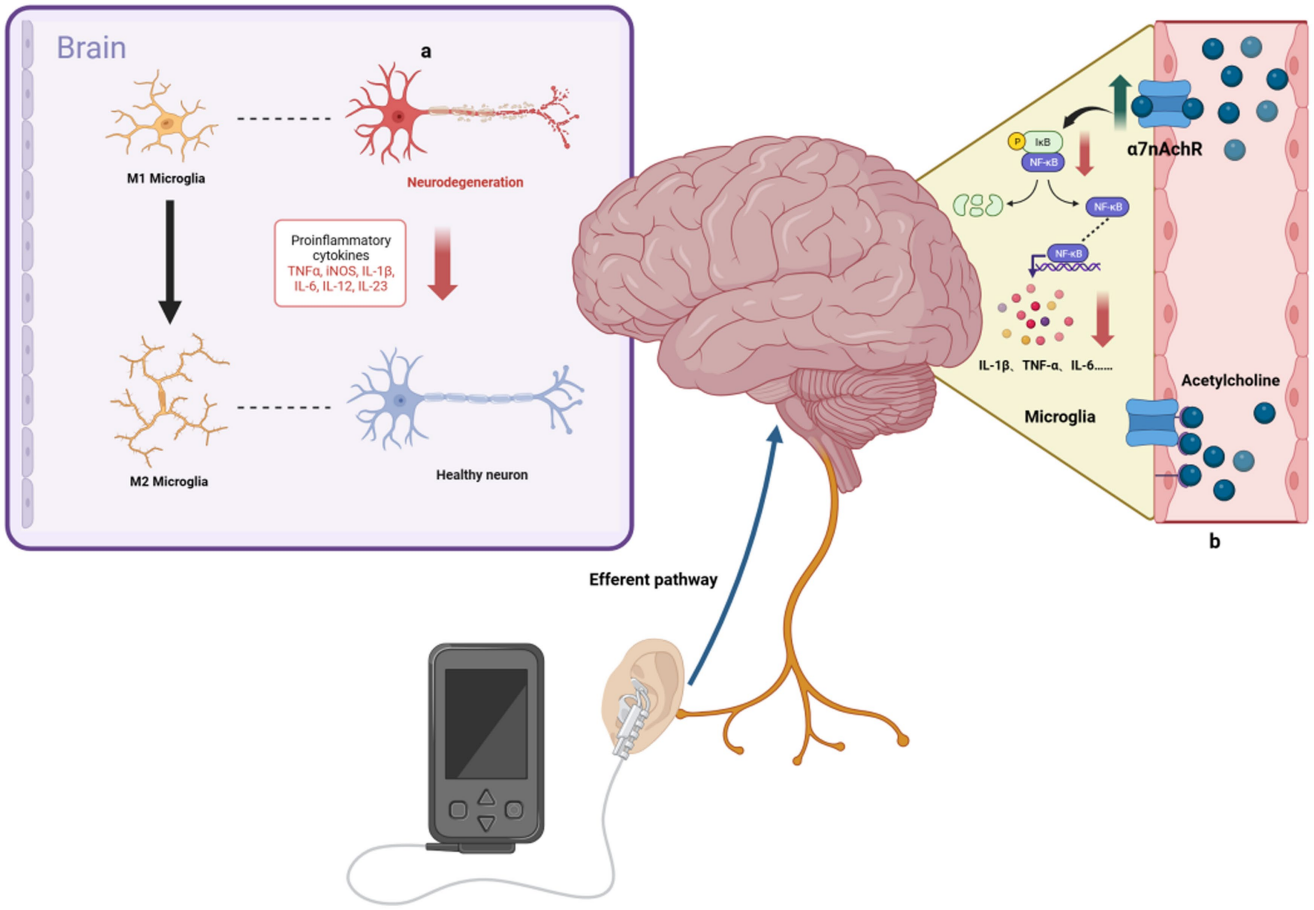
Anti-inflammatory mechanisms of taVNS. **(A)** taVNS induces microglia polarization toward an anti-inflammatory phenotype and reduces levels of pro-inflammatory factors, ameliorating hippocampal neuroinflammation. **(B)** By upregulating the anti-inflammatory effects of α7nAchR, taVNS has been shown to reduce the production of pro-inflammatory cytokines, such as IL-1β.

AD, a neurodegenerative disorder, is characterized by an inflammatory response in the CNS (Scheltens et al., 2021). The vital ability of tVNS to reduce inflammation would allow for fundamental progress in AD treatment (Figure 2). The α7 nicotinic acetylcholine receptor (α7nAchR) is vital to the VN’s anti-inflammatory effects (Wang et al., 2003), with VNS capable of modulating cholinergic anti-inflammatory pathways. This modulation inhibits cytokine release (e.g., TNF-α, IL-1β, IL-6), reducing inflammation in CNS regions such as the hippocampus (Namgung et al., 2022). tVNS, through the cholinergic anti-inflammatory pathway, similarly inhibits these inflammatory cytokines (Zhou et al., 2022) and has been observed to decrease plasma TNF-α levels and its expression in the PFC, hippocampus, and hypothalamus, thereby suppressing neurological inflammation (Guo et al., 2020).

Research by Wang et al. (2021) indicates that taVNS alleviates hippocampal neuroinflammation in rats via the α7nAchR/NF-κB signaling pathway, reducing IL-1β expression and inducing microglial phenotypic transformation. Additionally, Hou et al. (2023) found that tVNS reduces serum cytokine levels (TNF-α, IL-6, IL-1β, NF-κBp65) in a rat model of functional dyspepsia, an effect not seen post-vagotomy. The gut-brain axis is a bidirectional system linking the inflammatory responses of the gut and brain (Agirman et al., 2021; Aggarwal et al., 2022; Chen et al., 2022). VNS has been shown to effectively reduce intestinal inflammation, influencing ILs, TNF-α, and transforming growth factor β (TGF-β) levels (Sinniger et al., 2020). taVNS can alleviate symptoms of irritable bowel syndrome, such as abdominal pain and constipation, possibly by increasing vagal afferent fiber activity, as demonstrated by reduced serum TNF-α and IL-6 levels and plasma 5-HT levels (Shi et al., 2021). To sum-up, the ability of tVNS to modulate both the central and peripheral inflammation is particularly valuable (while underused yet) in the treatment of neurodegenerative disorders, as it was recently emphasized during the COVID-19 pandemic (Rangon et al., 2020; Borges et al., 2021).

## 5 VNS can re-orchestrate brain plasticity optimally in AD

VNS has been shown to upregulate the functional connectivity of the three core neurocognitive networks often impaired in AD (Li et al., 2019), i.e., the default-mode network (Putcha et al., 2022), the salience network (Zhang M. et al., 2022) and the central executive network (Daigle et al., 2022). A recent study by Keatch et al. (2023) suggests that tVNS shares similar central activation pathways with VNS. fMRI studies have demonstrated that taVNS can activate the LC and the NTS (Butt et al., 2020). Although imaging studies elucidating tVNS effects on AD are in the preliminary stages, some hypotheses have been formulated based on existing research. Although imaging studies elucidating tVNS effects on AD are in the preliminary stages, some hypotheses have been formulated based on existing research. A randomized crossover trial involving a healthy population found that taVNS influenced working memory by modulating the alpha band activity of the EGCs, potentially mediated by the GABA system (Konjusha et al., 2023). Huang et al. (2023) conducted a clinical study using fMRI and observed that repetitive taVNS could modulate functional connectivity within the CNS region of the vagal pathway and limbic system, including the bilateral hippocampus, postcentral gyrus, thalamus, and basal ganglia. During vagal stimulation, there was an increase in the amplitude of low-frequency fluctuation (ALFF) values in the lingual gyrus, fusiform gyrus, parahippocampal gyrus, and left middle temporal gyrus, correlating with improved memory and language functions (Zhang H. et al., 2022). Another study showed that taVNS amplified network connectivity between the hippocampus and regions associated with language learning (Murphy et al., 2023). These findings collectively suggest that vagal stimulation, including taVNS, can modulate the activity of brain regions related to cognition, particularly the hippocampus. Thus, it is plausible that taVNS could potentially improve clinical symptoms of AD, such as learning and memory function, by activating these brain regions.

**Figure 3:**
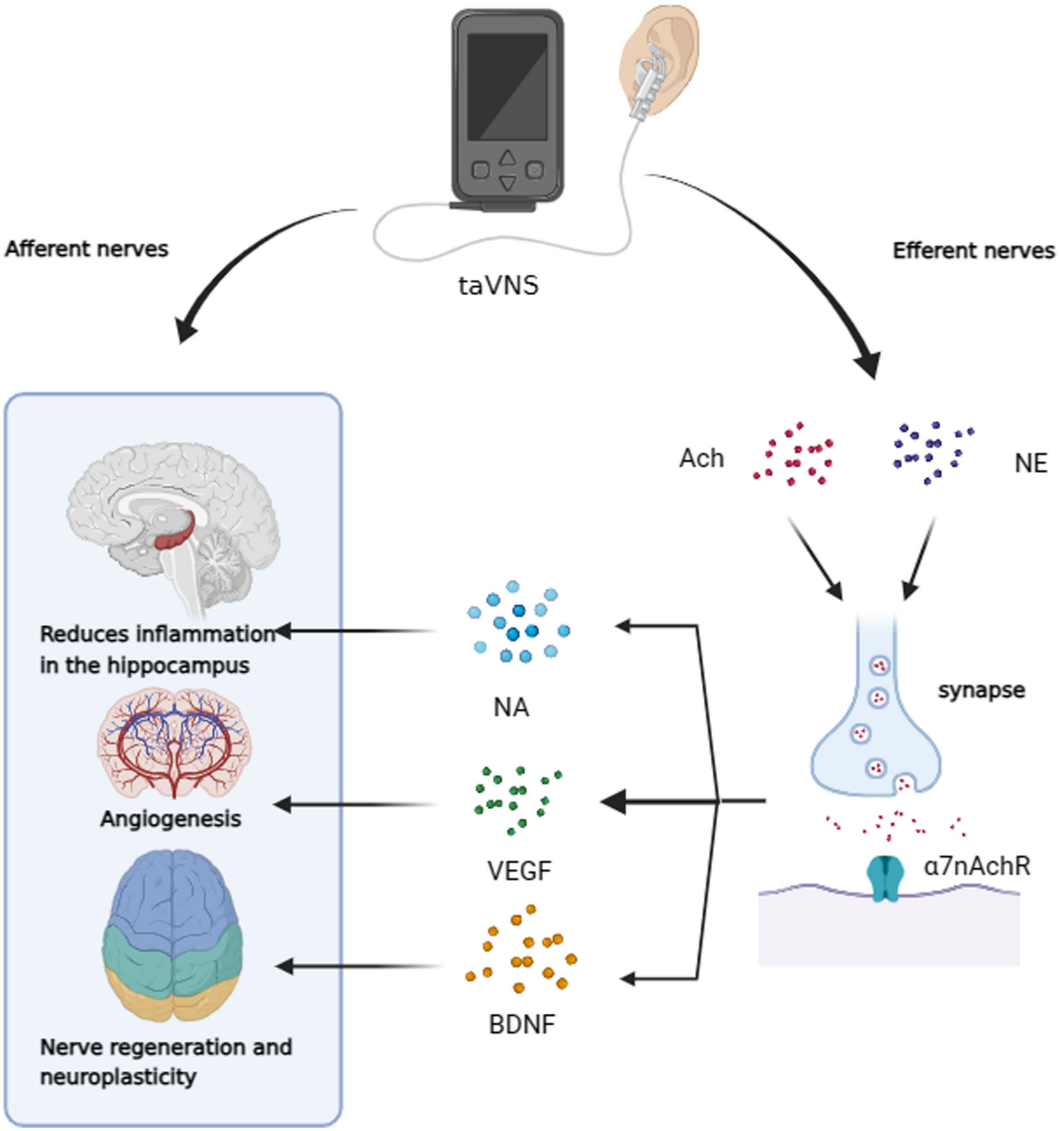
taVNS has the potential to improve cognitive dysfunction through afferent neuromodulation. It can also reduce hippocampal neuroinflammation, promote vascular regeneration, and enhance neuroplasticity through efferent nerves. This is achieved by increasing α7nAchR activity and releasing NA, VEGF, and BDNF.

VNS can drive the secretion of neurotransmitters and many other neurotrophic factors known to be important in AD (Figure 3). Gut microbiota, for instance, can increase the expression of brain-derived neurotrophic factor (BDNF) in the hippocampus via the VN (Amagase et al., 2023). Furthermore, taVNS has been reported to increase levels of vascular endothelial growth factor (VEGF) and BDNF in the brain (Long et al., 2022; Olsen et al., 2022). Furthermore, taVNS has been reported to increase levels of vascular endothelial growth factor (VEGF) and BDNF in the brain (Pihlaja et al., 2020), which has been shown to improve executive function in rodents (Grimonprez et al., 2015) and clinical performance in individuals with executive function deficits (De Taeye et al., 2014). Indirect markers of noradrenergic activity, such as P300, salivary alpha-amylase (sAA), and pupillary dilation, have been associated with VNS effects (Reimer et al., 2016; Privitera et al., 2021). taVNS has been demonstrated to stimulate NA release and enhance sAA secretion (Giraudier et al., 2022). In studies involving healthy participants, taVNS increased sAA levels post-intervention and mitigated the reduction in salivary cortisol concentration compared to sham-stimulated groups (Warren et al., 2019). Three of the six current studies have indicated that taVNS may regulate pupil dilation and reduce α oscillations in a dose-dependent manner during rest, supporting an NA mechanism hypothesis (Sharon et al., 2021; Urbin et al., 2021; D'Agostini et al., 2023). However, some studies do not support the idea that taVNS leads to changes in P3b in patients or noticeable activation of the LC (Borges et al., 2021; D'Agostini et al., 2022; Gadeyne et al., 2022). Additionally, a clinical trial did not observe changes in overall sAA levels as a result of taVNS, underscoring the need for more evidence to confirm the activation of the taVNS pathway through the NA release (Borges et al., 2021; D'Agostini et al., 2022; Gadeyne et al., 2022). Table 1 summarizes the characteristics found in these studies. The role of neurotransmitters in enhancing cognition through tVNS is crucial. While the NA pathway provides some evidence for the efficacy of tVNS in improving cognitive dysfunction in AD, conflicting results from various studies, possibly due to differences in stimulation parameters (Farrand et al., 2023) and individual variability, highlight the need for further validation and evidence.

**Table 1:** Association of taVNS with norepinephrine activity.

Study	Subjects of the study	Supports noradrenergic mechanisms	Result
D'Agostini et al. (2023)	Healthy human	Yes	taVNS enhances pupil dilation
Sharon et al. (2021)	Healthy human	Yes	taVNS induces pupil dilation and attenuation of occipital alpha oscillations
Urbin et al. (2021)	Healthy human	Yes	taVNS enhances pupil dilation
Gadeyne et al. (2022)	Healthy human	No	taVNS did not alter the P3b
Borges et al. (2021)	Healthy human	No	taVNS did not induces pupillary responses
Borges et al. (2021)	Healthy human	No	taVNS did not influence pupillary responses cortisol, and salivary alpha-amylase

## 6 Relationship between the vagus nerve and hypothalamus-pituitary–adrenal axis

The HPA axis is a critical component of the body’s response to psychological stress and has been involved in the pathogenesis of AD. This axis consists of a network involving the hypothalamus, pituitary gland, and adrenal gland, which collectively regulate the body’s stress response and directly influence the development of AD (Bisht et al., 2018). Chronic stress can alter brain function and structure relevant for Alzheimer pathology as old rhesus monkeys (mean age 25 years) who were exposed to chronic stress during their youth compared to control animals could be shown to accumulate a more than 7-fold higher density of ß-amyloid in temporal neocortex while synapse density in the same cortical areas was reduced by about 16% (Merrill et al., 2011). Clinical observations by Catania et al. (2009) revealed elevated cortisol levels in the basal ganglia of AD patients. Additionally, they observed that stress and glucocorticoids (GC) could lead to misprocessing of amyloid precursor peptides in the hippocampus and PFC, resulting in β-amyloid deposition, a hallmark of AD pathology. The gut microbiota is an important component of the BGM axis. The HPA axis also influences the gut microbiota, a complex ecosystem within the gastrointestinal tract consisting of bacteria, fungi, archaebacteria, viruses, and protozoa, in a symbiotic relationship with the host (Varesi et al., 2022). Research has linked changes in gut microbiota composition to the development of AD (Liu et al., 2020; Kim et al., 2022). Studies in mice have shown that long-term social stress can change the abundance and diversity of gut microbiota, dominated by *Bacteroide*s and *Lactobacillus* spp. genera while also increasing levels of interleukin-6 (IL-6) and monocyte chemoattractant protein-1 (MCP-1; Bailey et al., 2011; Galley and Bailey, 2014). Therefore, dysregulation of the HPA axis may lead to Aβ deposition and changes in the intestinal environment, contributing to the onset of neurodegenerative diseases, including AD.

Activation of the HPA axis can increase intestinal permeability, and conversely, stimulation of the VN inhibits HPA axis activation and improves hippocampal function. Prolonged stress reduces VN activity by stimulating the sympathetic nervous system, leading to increased gut permeability (Gareau et al., 2007; Taché and Bonaz, 2007). The VN also can activate exert anti-inflammatory effects through the cholinergic anti-inflammatory pathway (Bonaz et al., 2021). Recent research by Li et al. (2020) found that the electrical tVNS in the ear at 20 Hz reduced depression-like behavior in rats under mild stress. This intervention was associated with decreased HPA axis activity, as indicated by lower plasma levels of corticosterone (CORT) and adrenocorticotropic hormone (ACTH). The reduction in CORT also slowed hippocampal tissue atrophy and mitigated cognitive decline progression. Doerr et al. (2023) observed that salivary cortisol (sCort) levels decreased during taVNS in patients with temporal lobe epilepsy. This suggests that taVNS can modulate the stress response in epileptic patients through the HPA axis and the autonomic nervous system. Additionally, Hou et al.’s animal experiments showed that taVNS inhibited HPA axis activation, but this effect disappeared following vagotomy. Additionally, the animal experiments of Hou et al. (2023) showed that taVNS inhibited HPA axis activation, but this effect disappeared following vagotomy. In a clinical study, Gurel et al. (2020) found that patients with post-traumatic stress disorder (PTSD) who underwent 3 days of tcVNS had lower levels of plasma pituitary adenylate cyclase-activating peptide (PACAP) in the observation group compared to those in the sham-stimulation group. This indicates that tcVNS may help alleviate the neurobiological stress response in PTSD patients.

PACAP plays a vital role in maintaining the body’s stress response, and it can activate CRH to regulate the activity of the HPA axis, by stimulating CORT output (Mustafa, 2013). The HPA axis is a critical element of the BGM axis and exerts a bidirectional regulatory influence on AD progression. One possible mechanism could be the influence of tVNS on AD progression by modulating the function of the HPA axis through the VN.

## 7 Innovative strategies for treating Alzheimer’s disease with tVNS

The prevention and treatment of AD have emerged as a critical public health challenge globally. The BGM axis represents a promising therapeutic target for AD. Novel interventions based on this concept, such as probiotic therapy, microbiota transplantation, acupuncture, psychotherapy, and VNS, are currently being explored (YanLong et al., 2023).

The BGM axis is a complex bidirectional communication system that functions in both directions (Kowalski and Mulak, 2019). VNS can potentially modify—most of the time probably in anon-physiological way—the signal transmission, because we have no clue yet, how information is actually coded. It plays a crucial role in mediating the interaction between the CNS and the gut microbiota, including its by-products. Evidence suggests that inflammatory responses in the gut may increase intestinal barrier permeability, leading to the release of intestinal by-products such as short-chain fatty acids (SCFAs) and LPS into the systemic circulation, thereby affecting the CNS (Pellegrini et al., 2018; Doifode et al., 2021). The VN can help repair the intestinal barrier and modulate the composition of the intestinal microbiota through EGCs. Signaling via EGCs allows VNS, including tVNS, to influence the structure and function of the gut microbiota, promoting increased diversity. tVNS activates cholinergic anti-inflammatory pathways, improving the production of anti-inflammatory signals and reducing the levels of inflammatory factors such as TNF-α, IL-1β, and IL-6 in both the central and peripheral systems (Baig et al., 2022). Therefore, it is hypothesized that stimulating the VN can positively influence the BGM axis, repairing the abnormal intestinal environment. This action may prevent harmful signaling to the CNS, potentially improving cognitive dysfunction associated with AD. However, further research is needed to fully understand the impact of tVNS on the intestinal environment, including microbial species, barriers, and derivatives. Optimization of experimental protocols in this area remains a critical area of investigation.

Imaging studies have demonstrated that tVNS can activate specific brain regions associated with cognitive functions, including the LC, NTS, and hippocampus. These areas are crucial for cognitive processes, and the LC-NA pathway is particularly relevant in the pathogenesis of AD. Certain studies have identified activation of the NA pathway during tVNS, as evidenced by assays, including sAA and pupil dilation, supporting the potential of tVNS in treating cognitive impairments in AD. Therefore, VNS can potentially modify, often in a non-physiological manner, signal transmission within the BGM axis, which is integral to the physiological basis of AD. By modulating this communication, tVNS may have therapeutic effects on AD pathology. However, current research into tVNS for AD treatment is still in preliminary stages, and its efficacy is not yet widely recognized. These deficiencies are partly due to tVNS being a relatively new adjuvant therapy. To strengthen the clinical evidence supporting tVNS intervention in AD, future research should focus on conducting large-sample RCTs, multicenter clinical studies, and observational studies. In addition, preclinical research is also essential.

tVNS is capable of influencing the HPA axis, which is integral to the human stress response and an essential component of the BGM axis. The HPA axis plays a pivotal role in the pathogenesis of AD. Chronic stress can trigger microglial transformation into a pro-inflammatory phenotype, leading to an inflammatory response in the CNS. This response can alter the structure and function of the intestinal microbiota and impact the permeability of the intestinal barrier (Bisht et al., 2018; Tache et al., 2018). tVNS, through its cholinergic anti-inflammatory pathway, can modulate the HPA axis, impacting the functioning of the hypothalamus, pituitary, and adrenal glands. Initially developed and used for conditions such as depression and schizophrenia, the non-invasive VNS has shown promise in treating chronic stress, owing to its interaction with the VN’s properties and distribution. However, the exploration of the HPA axis within the BGM as a mechanism for understanding and treating AD has yet to be fully recognized. The potential of tVNS to treat AD through the BGM presents a unique opportunity, particularly in the context of current global health challenges. By focusing on the relationship between the HPA axis and AD, researchers can further elucidate the mechanisms by which tVNS may be effective in treating AD.

VNS, originally developed for treating epilepsy, has expanded its therapeutic scope with the advent of non-invasive stimulation techniques. taVNS, targeting the auricular branch of the vagus nerve, has gained popularity in clinical practice due to its non-invasive nature and ease of application. Emerging research indicates that tVNS can activate neurological and endocrine pathways similar to those stimulated by traditional VNS (Sharon et al., 2021; Urbin et al., 2021; D'Agostini et al., 2023). This evidence supports the potential use of tVNS as a therapeutic modality for neurodegenerative diseases like AD, offering a non-invasive and patient-friendly option for managing such conditions.

The effectiveness and mechanistic understanding of taVNS are complex and influenced by various parameters. Existing studies present conflicting evidence regarding the use of sAA and pupil dilation as indicators of NA activity, emphasizing the complexity of taVNS’s impact on physiological pathways. These discrepancies may arise from differences in intervention parameters and individual responses to taVNS. In clinical practice, taVNS mainly targets the external auricle (Badran et al., 2018), with common stimulation sites being the tragus and the cymba conchae of the anterior wall of the outer ear canal (tragus) and the cymba conchae, though the field is still identifying better stimulation sites. While left ear stimulation is generally preferred to avoid bradycardia (Baig et al., 2022), an extensive study by Badran et al. (2019) reports no adverse events with right ear stimulation. Stimulation parameters in taVNS vary across studies, as highlighted by Butt et al. (2020) in their review of taVNS-induced fMRI and somatosensory-evoked potential studies. Most taVNS applications use pulsed stimuli with pulse widths ranging from 250 to 500 μs and frequencies between 10 to 25 Hz. The current intensity is typically kept below 5 mA, tailored to individual tolerances. These variations in taVNS application underscore the need for further research to optimize stimulation sites, parameters, and individualized approaches to maximize therapeutic efficacy and safety, especially in the context of neurodegenerative diseases like Alzheimer’s Disease.

In taVNS interventions, managing current intensity is crucial for patient comfort and efficacy. Current intensities are typically set at or below the pain threshold, creating a perceptible yet tolerable sensation (Ellrich, 2019). Badran et al. (2019) highlighted the potential for optimizing taVNS, suggesting that while 25 Hz stimulation has shown promising outcomes, exploring higher frequencies, alternative stimulation sites, and duty cycle adjustments could enhance therapeutic effects. The therapeutic potential of gamma frequency (30–80 Hz) stimulation is further corroborated by other studies that combined multisensory (auditory and visual) stimuli at this frequency to improve AD pathology and cognition in rodent models of the disease (Iaccarino et al., 2018; Adaikkan et al., 2019; Martorell et al., 2019). The gamma-frequency oscillation slows down in the lateral entorhinal cortex (LEC) in AD mice. The LEC is the key structures relaying memory-related information between the neocortex and the hippocampus (Klein et al., 2016). Repeated presentations of sensory stimuli can generate transient gamma-frequency responses in the neocortex. These responses exhibit plasticity in a task-dependent manner (Ainsworth et al., 2016). A repeated auditory stimulation at 40 Hz effects across multiple cell types—neurons, microglia, astrocytes, and vasculature—and brain regions to drive the attenuation of AD-related pathology in a circuit-wide manner (Martorell et al., 2019). Adaikkan et al. (2019) stated that the preservation of neurons and synapses, reduced neuroinflammation, enhanced synaptic transmission and synaptic plasticity gene expression, and/or enhanced coherent gamma oscillations, may contribute to improvement of cognitive processes through Gamma ENtrainment Using Sensory stimuli. Iaccarino et al. (2016) demonstrate that optogenetic entrainment of neurons at gamma frequency can decrease Aβ levels by enhancing the phagocytic activity of microglia in the brain. Park et al. (2021) also reported positive outcomes with 40 Hz transcranial ultrasound stimulation in an AD mouse model. The NTS projects directly or indirectly to multiple brain regions, including the locus oeruleus, hypothalamus, thalamus, amygdala, hippocampus, and prefrontal cortex (Ter Horst et al., 1989; Van Eden and Buijs, 2000; Castle et al., 2005). Even though no evidence has been provided that tVNS directly induces gamma oscillations in the target brain regions, there is evidence that stimulating the vagus nerve has effects which are very similar to directly entraining brains with AD at frequencies in the gamma frequency range. This recommendation is supported by Yu et al. (2023) who demonstrated the benefits of higher-frequency stimulation. Their study found that 40 Hz taVNS inhibited hippocampal P2X7R/NLRP3/Caspase-1 signaling pathways and improved spatial learning and memory in 6-month-old APP/PS1 mice. These findings collectively suggest that frequencies higher than 40 Hz might be more effective for treating AD. Therefore, it is reasonable to suggest that repeated 40 Hz tVNS stimulation may induce gamma oscillations in brain regions such as the neocortex and hippocampus, thereby improving the reduced gamma frequency observed in AD and increasing information exchange between hippocampal-neocortex. Furthermore, 40 Hz tVNS may impact microglia, vascular regions, and nerve regeneration through repeated stimulation, driving the attenuation of Alzheimer’s pathology. However, it is doubtful whether gamma frequency oscillations induced by tVNS can be transmitted to the neocortex and hippocampus with minimal loss. Much rather, gamma rhythms in cortical structures follow mostly their intrinsic dynamic and therefore might only be triggered by faster signal modulation in their input.

Other related reviews have also focused on tVNS treatment for AD. Yap et al. (2020) also suggested in their critical review that despite the breadth of research being undertaken, many questions remain regarding the most effective stimulation sites and parameters. Many researchers in the current study used different assessment metrics, but there are major issues with inconsistent use of terminology and incomplete reporting when reporting the results of incomparable and non-reproducible experiments. The authors summarized the parameters used in tVNS, such as stimulation site, waveform, frequency and current intensity, and suggested that further research into the specific mechanism, stimulation parameters and durability of effects of tVNS should be conducted under a more standardized research protocol in the future. Vargas-Caballero et al. (2022) stated that VNS can target multiple mechanisms of Alzheimer’s disease with potential to modify the disease trajectory. They also pointed out that if recent trials of tVNS are unsuccessful or the effect sizes are too small to produce significant improvement, it may be necessary to re-evaluate the stimulation paradigms. Similar to the two reviews mentioned above, this review considers that future research should focus on fine-tuning the taVNS stimulation pattern, considering factors such as frequency, intensity, and sensory modalities to maximize its therapeutic potential for neurodegenerative conditions like AD. Furthermore, individual sensitivity for reacting to certain kinds of stimulation parameters should be explored more in the future. This is significant for the development and innovation of tVNS technology equipment.

Through systematic review of relevant studies on tVNS in relation to the BGM, AD, and cognitive function indicates that tVNS may modulate BGMA system signaling. This modulation, which influences the NTS function, gut microbiota, inflammation, brain plasticity, and the HPA axis, has the potential to alleviate or prevent cognitive dysfunctions associated with AD. Therefore, tVNS has emerged as a potentially cost-effective and effective therapeutic intervention for AD. Notably, recent research suggests that prefrontally modulated vagal neuroimmunomodulation can affect biological aging by influencing telomere length, supporting the potential of tVNS in mitigating AD (Ask and Sütterlin, 2022).

## Author contributions

LY: Formal Analysis, Methodology, Project administration, Software, Validation, Writing – original draft, Writing – review & editing. HL: Conceptualization, Formal Analysis, Methodology, Project administration, Validation, Writing – review & editing. YQ: Conceptualization, Formal Analysis, Methodology, Project administration, Validation, Writing – review & editing. JZ: Conceptualization, Writing – review & editing. SC: Conceptualization, Writing – review & editing. XZ: Conceptualization, Writing – review & editing. LW: Conceptualization, Writing – review & editing. YW: Funding acquisition, Writing – review & editing. MW: Conceptualization, Supervision, Writing – review & editing. TY: Funding acquisition, Supervision, Writing – review & editing.
